# Viral load suppression after intensive adherence counselling among adult people living with HIV at Kiswa health centre, Kampala: a retrospective cohort study. Secondary data analysis

**DOI:** 10.1186/s12981-023-00513-3

**Published:** 2023-03-30

**Authors:** Catherine Nakaye, Nelson Mukiza, Denis Mawanda, Hajira Kataike, Hellen Kaganzi, Grace Miriam Ahimbisibwe, Gerald Bright Businge, Raymonds Crespo Kyambadde, Rita Nakalega

**Affiliations:** 1 Makerere University-Johns Hopkins University (MU-JHU) Research Collaboration Kampala, Kampala, Uganda; 2 RineCynth Advisory Limited, Kampala, Uganda

**Keywords:** Viral suppression, Intensive Adherence Counselling, ART, HIV, Uganda

## Abstract

**Background:**

The Joint United Nations Programme on HIV/AIDS through the 95-95-95 target requires 95% of people living with HIV (PLHIV) on antiretroviral treatment (ART) to be virally suppressed. Viral Load (VL) non-suppression has been found to be associated with suboptimal ART adherence, and Intensive Adherence Counselling (IAC) has been shown to lead to VL re-suppression by over 70% in PLHIV on ART. Currently, there is data paucity on VL suppression after IAC in adult PLHIV in Uganda. This study aimed to evaluate the proportion of VL suppression after IAC and associated factors among adult PLHIV on ART at Kiswa Health Centre in Kampala, Uganda.

**Methods:**

Study was a retrospective cohort design and employed secondary data analysis to review routine program data. Medical records of adult PLHIV on ART for at least six months with VL non-suppression from January 2018 to June 2020 at Kiswa HIV clinic were examined in May 2021. Descriptive statistics were applied to determine sample characteristics and study outcome proportions. Multivariable modified Poisson regression analysis was employed to assess predictors of VL suppression after IAC.

**Results:**

Analysis included 323 study participants of whom 204 (63.2%) were female, 137 (42.4%) were between the age of 30 and 39 years; and median age was 35 years (interquartile range [IQR] 29–42). Participant linkage to IAC was 100%. Participants who received the first IAC session within 30 days or less after unsuppressed VL result were 48.6% (157/323). Participants who received recommended three or more IAC sessions and achieved VL suppression were 66.4% (202/304). The percentage of participants who completed three IAC sessions in recommended 12 weeks was 34%. Receipt of three IAC sessions (ARR = 1.33, 95%CI: 1.15–1.53, p < 0.001), having baseline VL of 1,000–4,999 copies/ml (ARR = 1.47, 95%CI: 1.25–1.73, p < 0.001) and taking Dolutegravir containing ART regimen were factors significantly associated with VL suppression after IAC.

**Conclusion:**

VL suppression proportion of 66.4% after IAC in this population was comparable to 70%, the percentage over which adherence interventions have been shown to cause VL re-suppression. However, timely IAC intervention is needed from receipt of unsuppressed VL results to IAC process completion.

**Supplementary Information:**

The online version contains supplementary material available at 10.1186/s12981-023-00513-3.

## Introduction

According to UNAIDS, 95% of PLHIV on ART should be virally suppressed as was ambitiously set through United Nations’ 95-95-95 target [1] fast tracking the sustainable development goal to end the HIV epidemic by 2030 [2, 3]. In 2020, out of 37.7 million PLHIV globally, 73% had access to ART and 66% had achieved viral suppression [4]. In Uganda by 2020, out of the 1.4 million PLHIV, 91% knew their HIV status, 90% were on ART and 82% had viral suppression [5]. In order to achieve benefits of early ART initiation, PLHIV on ART must be virologically suppressed [6, 7], which hinges on effective behavior change interventions by HIV care providers, health sector leadership and PLHIV themselves to facilitate good adherence to ART [8]. Good ART adherence defines as properly following treatment provider recommendations regarding ART dosage, frequency and timing of swallowing medication [9]. Poor ART adherence is a major cause of treatment failure in PLHIV [10–14] and ensuring sustained adherence to ART can achieve viral suppression except for presence of HIV drug resistance [15–20]. If a person living with HIV is not virally suppressed, they will have slow immune system recovery, HIV disease advancement, increased morbidity and an enhanced HIV infection transmission risk [2, 21–24]. ART-associated side effects, younger age, substance use, depression and forgetting dosing time are linked to sub-optimal ART adherence [14, 25]. Additionally, individual ART initiation incentive, education level, duration on ART, stigmatization and HIV status disclosure affect ART adherence [19, 25, 26].

IAC assists individuals to develop a comprehensive ART adherence strategy by identifying and gaining insight of adherence barriers, explore practical ways to overcome the barriers and generate an ART adherence plan [13, 18].

WHO endorses IAC provision for both children and adults who are found to have an unsuppressed VL, which adherence support has been shown to improve the VL suppression by up to 70% [17, 18]. HIV VL suppression achieved through IAC ensures extended preservation of first-line ART regimens and reduces HIV resistant strains in novel infections [12]. PLHIV at increased likelihood of suboptimal or non-adherence to ART profit from focused adherence interventions that are contextual, socially acceptable and hinged on principles of human reason and behavioral values [27, 28]. A multidisciplinary team of counselors, clinicians, nurses and peers provides IAC to clients according to set guidelines by ministry of health for offering IAC using the 5 As counseling framework of Assess, Advise, Assist, Agree on and Arrange [13, 18].

The Uganda Ministry of Health guidelines state that PLHIV on ART with un-suppressed VL should receive IAC, one counseling session monthly for three months, followed by repeat VL measurement a month after the third IAC session [13]. Virally suppressed individuals on repeat VL testing continue with the same ART regimens while those with unsuppressed VL are considered for a switch to the next line ART regimens [13]. Effective and timely IAC intervention is necessary for individuals with unsuppressed VL to achieve viral suppression and maximally benefit from ART [17]. Delayed confirmation of unsuppressed VL and late linkage of individuals with unsuppressed VL to the IAC intervention contribute to late detection of ART failure [12]. A need to re-evaluate the IAC intervention implementation among virally non-suppressed PLHIV on ART has already been documented for Uganda [16, 29]. Necessity to improve the delivery of IAC with component structuring, context and information packaging has been expressed as well [30].

Although Uganda has employed the IAC intervention program since 2015, scanty research data is available on HIV viral suppression after IAC. Central Uganda has higher HIV prevalence compared to other regions of the country; and urban areas have an even greater HIV burden than rural areas with urban areas HIV prevalence being 7.5% in comparison to 5.8% in rural areas [31]. There is paucity of data on proportions of HIV VL suppression after IAC and its predictors for adult PLHIV with unsuppressed VL in an urban setting in Uganda, which data is beneficial in critical appraisal of IAC implementation in Kampala, an overly populated urban center with a viral suppression prevalence of only 62.1% [31].

This study aimed to evaluate the proportion of VL suppression after IAC and associated factors among adult PLHIV on ART at Kiswa Health Centre in Kampala, Uganda.

## Materials and methods

### Study design and setting

A retrospective cohort study design that employed approaches of secondary data analysis of routinely available program data for PLHIV receiving ART at Kiswa Health Centre, a public HIV clinic in Kampala district was used. The proportion of PLHIV with VL greater or equal to (≥) 1000 copies/ml, on a test done between January 2018 and June 2020, who achieved VL suppression following IAC was determined; as well as the time to completion of the IAC sessions and the factors that were associated with VL suppression after IAC.

The study was conducted in Kampala district at an HIV Clinic in Kiswa Health Centre situated within Nakawa Division. Located in the central region, Kampala, the capital city of Uganda is the second most populated district in Uganda with a population size of 1,507,080 people, 51.9% being females [32]. Kampala is 100% urban and remains the most populated urban center in Uganda [32].

According to a Uganda national survey conducted between August 2016 and March 2017, Kampala capital city had an HIV prevalence of 6.9% amongst people aged 15 to 64 years [31]. Kiswa Health Centre was carefully selected because of its urban location and easy accessibility as one of the HIV clinics under Kampala Capital City Authority within Kampala district.

### Standard of care ART at time of study conduct

As of February 2020 under the guidance of the Uganda Ministry of Health, regimens containing Dolutegravir (DTG) were recommended for use as the preferred first and second-line treatment regimens for all HIV infected clients because of existence of great levels of HIV resistance to non-nucleoside reverse transcriptase inhibitors (NNRTI) containing firstline ART regimens and superior efficacy of DTG containing regimens compared to EFV based ART regimens [13]. Additionally, DTG was documented to be better tolerated than EFV and had a high genetic barrier. Therefore, Tenofovir Disoproxil Fumarate (TDF)/ Lamivudine (3TC) and Dolutegravir (DTG) became the recommended firstline ART regimen for adolescents and adults starting ART and having a body weight of ≥ 30 kg in Uganda in February 2020 [13]. Transition to DTG containing ART regimen was also done for all eligible PLHIV who were already on ART. PLHIV ineligible for DTG due to being diabetic or having risk factors for hyperglycemia received Efavirenz or Atazanavir (ATV/r) containing ART regimens as firstline ART. As per the ministry of health guidelines, clients are seen monthly in the clinic for the first three months following ART initiation and receive monthly ART refills. Subsequently, if the client is clinically well, they are given three monthly clinic appointments with 3 months ART refills. The first viral load monitoring is performed six months following ART initiation. If client is virally suppressed, the next viral load is done 12 months after ART initiation and subsequent viral load monitoring is done yearly there after.

### Population and procedures

The study population was all PLHIV aged 18 years or older, who had been on ART for at least six months with VL ≥ 1000 copies/ml, on a test done between January 2018 and June 2020. Data collection was completed in four weeks in May 2021.

Anonymized client information was used for secondary analysis and each study participant received a unique identifier. Data on key variables of interest including age in years, sex, level of education, occupation, religion, marital status, WHO clinical stage for HIV infection, ART regimen at time of VL non-suppression, ART regimen initiation date, CD4 cell count at time of VL non-suppression, non-suppressed VL test result at beginning of IAC, number of IAC sessions received, dates IAC sessions were received and repeat VL test result following IAC was abstracted from the HIV clinic source records and captured into a Microsoft® Excel database. The primary sources of data were the HIV clinic’s health management information system, ART registers, VL registers, IAC registers and client treatment files/cards. Data was thoroughly reviewed to ensure quality.

### Laboratory methods

VL testing is done at the Central Public Health Laboratories and VL test results received from the national laboratory are recorded in VL registers and client cards by health facility staff and given to the clients at subsequent clinic visits. The CD4 counts are measured in the HIV clinic laboratory. The quality control (QC) procedure for flow cytometry is done every beginning of day before any samples are run. The QC report obtained is compared to expected value range and any measures obtained that are out of range are checked again through procedure re-run. CD4 testing is not performed if expected QC measures are not obtained. The QC review form log is completed for each QC procedure done and is reviewed by the QC supervisor or designee on a daily basis.

### Definition of adequate IAC

As per the Uganda ministry of health consolidated guidelines for the prevention and treatment of HIV and AIDS, adequate IAC is considered as having a minimum of three consecutive IAC sessions following a non-suppressed viral load [13]. Therefore, a participant was considered as having had adequate IAC when they had documented three or more IAC sessions following a baseline non-suppressed viral load.

### Loss to follow up and missing information

An individual was considered lost to follow up when he or she did not return to the HIV clinic for a visit in three or more consecutive months at any time point in their HIV care following ART initiation as per the Uganda Ministry of Health guidelines [13]. Clients with missing information are those who did not have a documented repeat viral load result after any number of IAC sessions provided following a baseline non-suppressed viral load. The repeat viral load result was found missing for such clients because either client did not return to the HIV clinic for repeat testing or the repeat VL results were not yet available at time of data collection.

### Statistical analysis

The primary study outcome was VL suppression after IAC. VL suppression was defined as a VL result of less than 1000 copies/ml [13, 18]. A VL test result equal to or greater than 1000 copies/ml was termed as VL non-suppression. The time to first IAC session was defined as the time difference between the date of the baseline non-suppressed VL test result and date of first IAC session. Data was exported to Stata version 14 computer software package for analysis. The proportions of study participants with VL ≥ 1000 copies/ml who received IAC and individuals who achieved VL suppression following an adequate IAC intervention were determined. Additionally, the time taken to completion of the IAC sessions was described, as well as the factors associated with VL suppression after IAC in the study population. Descriptive statistical analysis was performed for the socio-demographic and clinical characteristics of the study participants using means and frequencies. Bi-variable analysis was used to analyze the relationship between each independent variable and the primary outcome.

P-values of less than 0.05 were considered statistically significant [33, 34]. All variables in the bivariable analysis that had a p-value < 0.2 for association with VL suppression after IAC were included in the multivariable modified Poisson regression analysis to assess independent relationships further while adjusting for potential confounders [33–35]. At multivariable analysis, p-values ≤ 0.05 were considered statistically significant. Time to completion of the IAC sessions was expressed by the Kaplan–Meier survival curve [34, 36].

### Ethics statement

Local ethical approval was provided by Mildmay Uganda Research Ethics Committee (1012–2020). Additionally, a waiver of consent was attained from the same IRB since retrospective study of de-identified data was to be done. Study administrative clearance was received from Kampala Capital City Authority and the In-Charge of Kiswa Health Centre III. The raw data will be retained for five years. Anonymized data and lack of access to participant files offered protection from risk of confidentiality breach.

## Results

**Fig. 1:**
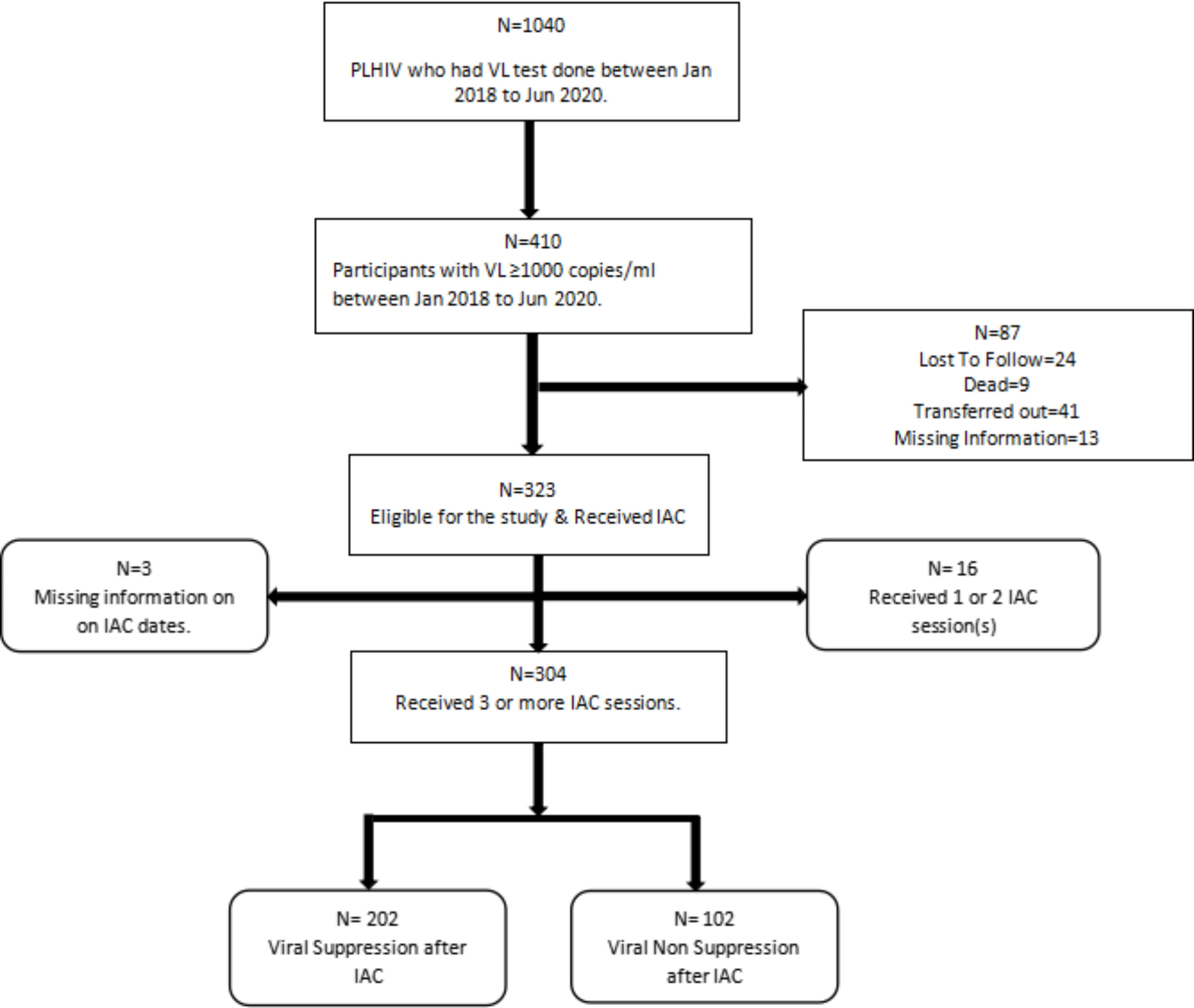
Illustration of the study profile

A total of 410 adult PLHIV at Kiswa Health Centre had an unsuppressed viral load ≥ 1000 copies/ml in the period from January 2018 to June 2020. The number of eligible study participants was 323 (79%) as 87 (21%) individuals were either lost to follow, transferred out, dead or had missing information and these were not included in analysis (Fig. 1).

### Baseline characteristics of study participants

Out of the 323 participants, 204 (63.2%) were female. A considerable number of study participants; 137 (42.4%) were between the age of 30 and 39 years. The median age was 35 years (IQR: 29–42). Most participants (65.3%) were from Kampala district. The median time duration on ART at the time that the participant had a detectable VL was 4 years (IQR: 1–7), with the shortest time on ART being 6 months while the longest duration on ART was 16 years. The number of the study participants who were on an Efavirenz (EFV) based ART regimen was 143 (44.3%). Out of 143 participants who were on an EFV based regimen, 115 were taking Tenofovir Disoproxil Fumarate (TDF)/ Lamivudine (3TC)/ Efavirenz (EFV); 27 were taking Zidovudine (AZT) / Lamivudine (3TC)/ Efavirenz (EFV) while one participant was taking Abacavir (ABC)/ Lamivudine (3TC)/ Efavirenz (EFV). Additionally, majority of the participants were in WHO clinical stage 1; 253/266 (95.1%) and had been on ART for more than 5 years at time of VL detection; 153 (47.8%).

### IAC: number of sessions received and process completion timelines

All 323 study participants received at least one IAC session following the baseline unsuppressed VL result. The majority of participants; 208 (64.4%) received more than three IAC sessions while 99 (30.7%) participants received three IAC sessions. Participants who had one or two IAC sessions were 16.

The time to IAC completion was determined as the time difference from the date of the baseline non-suppressed VL test result to the last documented offered IAC session as per the clients’ records prior to subsequent repeat evaluation of viral load. Only 34% of study participants completed the IAC intervention in 12 weeks while 46.7% of participants completed IAC between 13 and 24 weeks. The recommended time duration within which the three standard IAC sessions should be completed as per the Ministry of Health guidelines is 12 weeks [13].

**Fig. 2:**
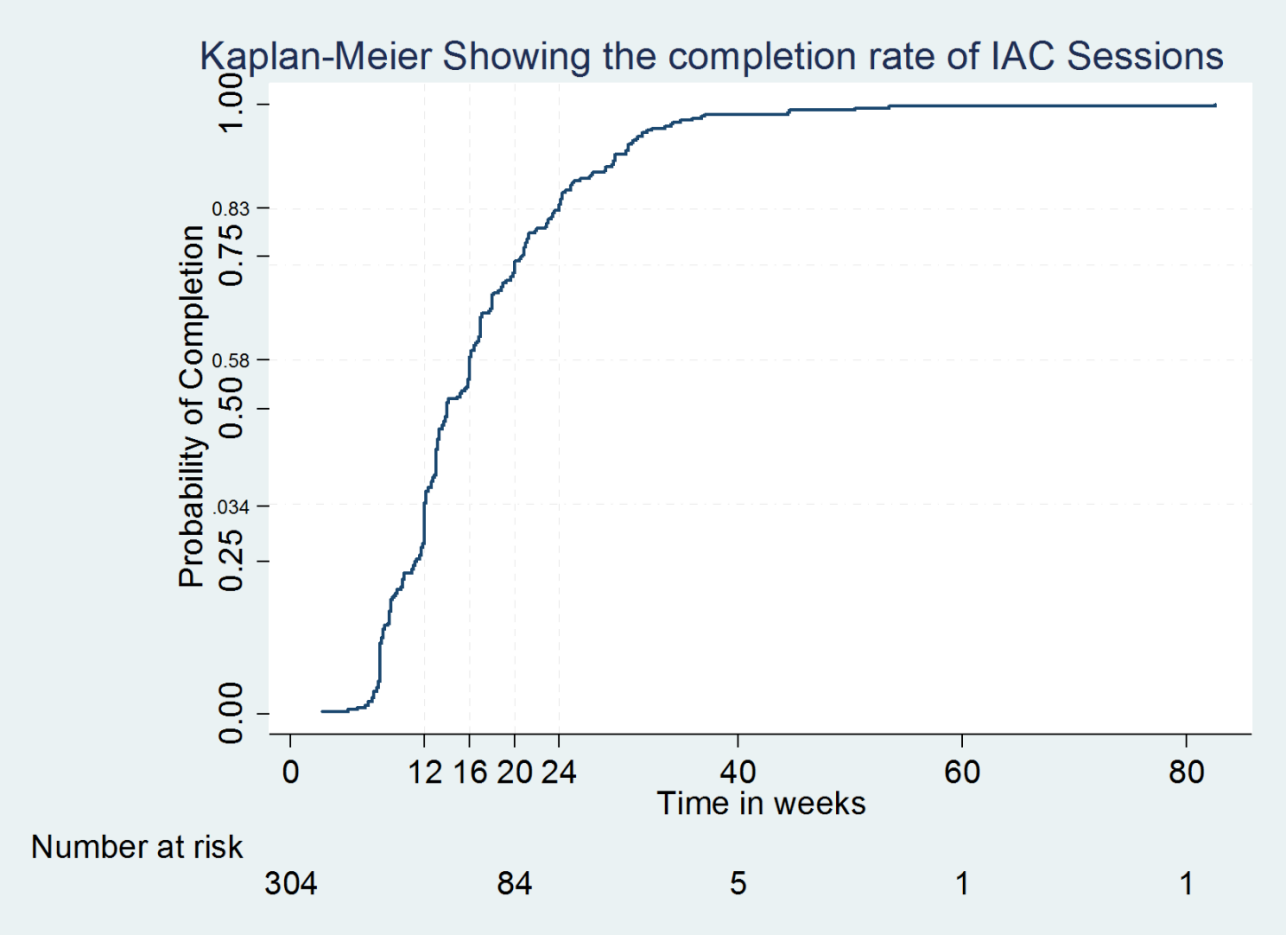
Kaplan–Meier survival curve showing the completion rate of IAC sessions in the study population of participants who received three or more sessions

For this analysis, only study participants who received the recommended three IAC sessions or more (304) were included as these were considered to have completed the IAC intervention. Out of 323 participants, 16 had either one [7] or two [9] IAC sessions and three participants had missing IAC dates for the sessions recorded and hence these were excluded from the analysis. At 12 weeks (3 months), 34% of the participants had completed IAC, 58% had completed IAC at 16 weeks (4 months), 83% had completed at 24 weeks (6 months) and the remaining 17% completed IAC in more than six months (Fig. 2).

### VL suppression after IAC and associated factors

Out of the 304 study participants with baseline viral non-suppression who received adequate IAC, 202 (66.4%) achieved VL suppression after the intensive adherence counseling intervention. (95% CI: 60.9-72.6%). At multivariable analysis, study participants who received three IAC sessions were 33% more likely to have VL suppression than those who received more than 3 IAC sessions (4, 5 or 6 sessions), ARR: 1.33 (1.15–1.53), p < 0.001. Similarly, participants who had baseline VL of 1,000–4,999 copies/ml were 47% more likely to virally suppress compared to those with VL ≥ 10,000 copies/ml, ARR: 1.47(1.25–1.73), p < 0.001. Lastly, participants on DTG containing ART regimens were 61% more likely to have viral suppression when compared to those on EFV based ART regimen, ARR: 1.61 (1.37–1.88), p < 0.001.

## Discussion

In this retrospective cohort study, the HIV VL suppression proportion was 66.4% following adequate IAC intervention for adult PLHIV at Kiswa Health Centre, who had been on ART for at least six months and had an unsuppressed VL in the period from January 2018 and June 2020. Linkage to IAC following the baseline unsuppressed VL was 100% in the study population. The percentage of study participants who were linked to the IAC intervention in less than a month from the time of non-suppressed VL result was 48.6%. The percentage of study participants who received three or more IAC sessions was 95% and only 34% completed the IAC intervention in the recommended 12 weeks.

A retrospective cohort study in rural Uganda in 2017 found that 19% of 411 PLHIV with an unsuppressed VL did not receive IAC [37] while another earlier retrospective review conducted at 15 Uganda public health centers from June 2015 to December 2016 found that 7% of 449 study participants with VL above 1000 copies/ml did not have any IAC session provided [16]. The observed improvement in IAC linkage from our study reflects the increased efforts by Ministry of Health in Uganda which is geared towards increased routine HIV VL testing to quickly refer for the IAC intervention all PLHIV identified to have unsuppressed VL.

The study results on IAC linkage are comparable to findings from Ethiopia where all 235 PLHIV with unsuppressed VL involved in the retrospective cohort study received IAC [12], and findings from South Africa where all 400 eligible participants received IAC in a prospective cohort study [20]. IAC linkage in our study is 24.3% higher than the findings from a retrospective cohort study in Zimbabwe where out of 646 participants with unsuppressed VL, 75.7% were enrolled into IAC [14].

In this study cohort, 77.4% of study participants received the first IAC session within 60 days or less from the time of the baseline non-suppressed VL result. This is an improvement by approximately 20 weeks in the aptness of IAC intervention provision in a public health facility setting when compared to earlier findings where the first IAC session was received up to 200 days following the unsuppressed VL result availability by 75% of study participants [16]. However, the recommended time to first IAC session is 30 days as per Uganda national guidelines [13]. Enhanced adherence counseling (EAC) is more timely in Ethiopia as 8 weeks is the median time to the first counselling session [12].

Only 34% of participants completed the recommended three IAC sessions in 12 weeks in the study, a proportion 12.8% smaller than the proportion of study participants who completed IAC in the recommended three months in a retrospective cohort study in Ethiopia [12].

The percentage of study participants who completed the IAC intervention in 20 weeks was 75%, which was an improvement by 30 weeks in comparison to the findings of the same percentage (75%) of participants who finished the three IAC sessions after 50 weeks in an earlier retrospective cohort study conducted in Uganda [16].

The VL suppression proportion of 66.4% for all participants who received the recommended three or more IAC sessions is higher than 9%, the VL suppression percentage after IAC documented by a prospective cohort study [29] and higher than the VL suppression proportion of 23% documented by a retrospective research [16], which studies were both conducted in public HIV health care settings in Uganda. This VL suppression proportion of 66.4% is comparable to 70%, a percentage above which adherence support has been found to enhance VL suppression in PLHIV with a previously unsuppressed VL [17, 18]. In addition, 66.4% is comparable to: 66.4%, the VL suppression proportion after IAC for Ethiopia [12] and 67.5% [38] as well as 64% [20], the VL suppression proportions after IAC documented for South Africa. The VL suppression proportion of 66.4% found in our study is also comparable to 67%, the VL suppression proportion after IAC for Burkina Faso, Côte d’Ivoire, Senegal and Mali [39]. However, 66.4% is way higher than the VL suppression percentage stated in Zimbabwe following EAC [14] and the VL suppression of 10% found in a prospective cohort study in Tanzania following reinforced adherence counseling [40]. All study participants who achieved viral re-suppression after IAC did not require ART regimen switch which inherently helped in the preservation of the next line ART medications for when their need is warranted; a very core aim of the IAC intervention.

The study findings are contrary to those in a Swaziland study where EAC did not increase the likelihood of VL suppression [41].

Multivariable modified Poisson regression analysis findings demonstrated significant associations between VL suppression after IAC and number of IAC sessions received, baseline non-suppressed VL result and ART regimen type. The participants who received three IAC sessions were 33% more likely to have VL suppression than those who received more than 3 IAC sessions (4, 5 or 6 sessions), (ARR: 1.33, 95% CI: 1.16–1.53, p < 0.001). Individuals received more than three sessions of IAC if their adherence level was less than 95% on pill count at three consecutive IAC sessions as per the Ministry of health guidelines [13]. This includes clients with average adherence (85–94%) if two to four doses are missed in one month for once daily ART dosing or if four to eight doses are missed in one month for twice daily ART dosing on pill count [13]. More than three IAC sessions were also received by clients with documented poor adherence (< 85%) if ≥ five doses are missed in one month for once daily ART dosing or if ≥ nine doses are missed in one month for twice daily ART dosing on pill count [13]. Study participants who received more than three IAC sessions could have had more long standing adherence barriers to ART that poorly affected their viral load suppression and required adherence support for extended periods as compared to clients who had only three IAC sessions.

On the contrary, number of intensified adherence counseling sessions received was found not to be independently associated with VL suppression in a retrospective cohort study in Zimbabwe [14]. However in this Zimbabwean study, the participants who received three IAC sessions were more likely to virally suppress after IAC (68%) when they were compared to those that did not receive any enhanced adherence counseling session [14].

Study findings support the current Uganda national guidelines that the recommended three IAC sessions are potent and by a larger proportion, in reversing HIV viral non-suppression among PLHIV who have been on ART for at least six months [13], as it is mostly challenges with good adherence to ART that lead to VL non-suppression [10–14].

The study participants whose VL was in the range 1,000–4,999 copies/ml were 47% more likely to suppress after IAC compared to their counterparts with VL ≥ 10,000 copies/ml (ARR: 1.47, 95% CI: 1.25–1.73, p < 0.001). The study results are comparable to findings of a retrospective cohort study in Ethiopia where the baseline VL result was an important predictor of VL suppression after IAC [12]. In Ethiopia, the VL suppression probability was 56% lower for study participants with a VL greater than 10,000 copies/ml; and 7% lower for participants whose VL was between 5001 and 10,000 copies/ml when both categories were compared to those who had a baseline VL of 1,000–5,000 copies/ml [12]. Baseline VL ≥ 10,000 copies/ml was also associated with increased odds of VL non-suppression after EAC in Ethiopia from a case-control study [42]. Nevertheless, younger age, extended duration on ART, CD4 cell count of 201to 500/mm3 and residing in an urban area are factors found to be positively associated with suppression of VL after EAC in Ethiopia [43]. Similarly, in a retrospective cohort study in Zimbabwe, the participants with a baseline VL greater than 5000 copies/ml had a lower probability of VL suppression after IAC in comparison to those with baseline VL between 1000 and 5000 copies/ml, with the likelihood of VL suppression reducing with increasing VL test result [14]. The reduced likelihood of viral suppression after the IAC intervention in persons with very high baseline VL results can be linked to possible unidentified accumulated pre-existing resistance to ART and in such individuals, only switching to the next effective ART regimen most accurately determined by resistance testing can reverse the viral non-suppression. Unfortunately, due to cost implications, HIV resistance testing is reserved for limited categories of PLHIV in Uganda including those failing on their second and third line ART regimens [13].

### Study strengths and limitations

Study strengths include the cohort design which inherently provided a temporal causal relationship between IAC and VL suppression [44], a large sample size and utilization of routine patient information collected in a public HIV clinic in an urban setting, thus making the sample representative of the HIV clinic and generalizable to other urban HIV clinics in Kampala and Wakiso districts in Uganda. The major study limitation was utilization of routine clinic data which had missing information and therefore data analysis and interpretation was limited to routinely collected and documented variables in the client records. Important patient variables which could have affected the baseline and repeat VL testing, linkage to IAC and viral suppression post IAC like socio-economic status, education level and distance of patients’ residence to the HIV clinic were unavailable. Lastly, since it was an entirely quantitative study, health workers and participants’ experiences and perceptions regarding the IAC intervention and its delivery were not studied, which could have been examined by qualitative methods.

## Conclusion

In conclusion, client linkage to IAC was 100% and VL suppression after adequate IAC was high (66.4%) and comparable to 70%, a percentage above which adherence support has been found to enhance VL suppression in PLHIV with a previously unsuppressed VL [17, 18]. The percentage of study participants who received their first IAC session within the recommended 30 days or less after the unsuppressed VL result was 48.6%. Only 34% of participants completed the recommended three IAC sessions in 12 weeks as per the Uganda IAC guidelines [13]. Number of IAC sessions received by the participants, baseline non-suppressed VL test results and receiving a Dolutegravir based ART regimen were the factors found to be significantly associated with VL suppression after IAC. Qualitative research to understand perceptions surrounding the IAC intervention is critical and highly recommended.

**Table 1:** Socio-demographic and clinical characteristics of study participants

	Frequency	Percentage (%)
**Age in years**		
18-29	83	25.7
30-39	137	42.4
≥ 40	103	31.9
**Sex**		
Male	119	36.8
Female	204	63.2
**Participant address**		
Kampala	211	65.3
Wakiso	86	26.6
Others	26	8
**Marital status**		
Married	184	48.8
Unmarried	193	51.2
**Treatment Line (n=282)**		
First line	143	50.7
s line	139	49.3
**Who clinical stage at time of VL non-suppression (n=266)**		
Stage 1	253	95.1
Stage 2	6	2.3
Stage 3	3	1.1
Stage 4	4	1.5
**Duration on ART at Viral Load non-suppression (n=320)**		
< 1 year	64	20
> 1 to 5 years	103	32.2
> 5 years	153	47.8
**Non-suppressed viral load test results (Copies/ml)**		
1,000-4,999	148	45.8
5,000-10,000	35	10.8
≥ 10,000	140	43.3
**ART regimen at time of VL non-suppression**		
EFV based regimen	143	44.3
NVP based regimen	61	18.9
ATV/r based regimen	36	11.1
DTG based regimen	64	19.8
LPV/r based regimen	19	5.9

**Table 2:** ART regimens at time of baseline non-suppressed viral load

ART REGIMEN AT TIME OF VL NON-SUPPRESSION	Freq.	Percent	Cum.
ABC-3TC-ATV/r	1	0.31	0.31
ABC-3TC-EFV	1	0.31	0.62
ABC-3TC-LPV/r	2	0.62	1.24
AZT-3TC-ATV/r	9	2.79	4.02
AZT-3TC-DTG	1	0.31	4.33
AZT-3TC-EFV	27	8.36	12.69
AZT-3TC-LPV/r	4	1.24	13.93
AZT-3TC-NVP	49	15.17	29.10
TDF-3TC-ATV/r	26	8.05	37.15
TDF-3TC-DTG	63	19.50	56.66
TDF-3TC-EFV	115	35.60	92.26
TDF-3TC-LPV/r	13	4.02	96.28
TDF-3TC-NVP	12	3.72	100.00
Total	323	100.00	

**Table 3:** Bivariable analysis

Characteristic	Total (%)	Non- Suppressed (%)	Suppressed (%)	RR (CI95%)	P value
**Age in years**					
18–29	78 (25.6)	24 (30.8%)	54 (69.2%)	1	
30–39	130 (42.8)	47 (36.2%)	83 (63.8%)	0.92 (0.76–1.12)	0.420
≥ 40	96 (31.6)	31 (32.3%)	65 (67.7%)	0.98 (0.80–1.20)	0.830
**Sex**					
Female	189 (62.2)	70 (37.0%)	119 (63.0%)	1	
Male	115 (37.8)	32 (27.8%)	83 (72.2%)	1.15 (0.98–1.34)	0.090
**Participant address**					
Kampala	198 (65.1)	66 (33.3%)	132 (66.7%)	1	
Wakiso	81 (26.7)	28 (34.6%)	53 (65.4%)	0.98 (0.81–1.18)	0.844
Others	25 (8.2)	8 (32%)	17 (68%)	1.02 (0.77–1.36)	0.892
**Marital status (n = 268)**					
Married	140 (52.2)	47 (33.6%)	93 (66.4%)	1	
Unmarried	128 (47.8)	42 (32.8%)	86 (67.2%)	1.01 (0.85–1.20)	0.895
**Treatment line**					
First line	255 (83.9)	87 (34.1%)	168 (65.9%)	1	
s line	49 (16.1)	15 (30.6%)	34 (69.4%)	1.05 (0.86–1.29)	0.622
**WHO clinical stage at time of VL non-suppression (n = 253)**					
Stage 1	241(95.2)	78 (32.4%)	163 (67.6%)	1	
Stage 2	6(2.4)	2 (33.3%)	4 (66.7%)	0.99 (0.56–1.75)	0.961
Stage 3	3(1.2)	2 (66.7%)	1 (33.3%)	0.49 (0.10–2.46)	0.388
Stage 4	3(1.2)	1 (33.3%)	2 (66.7%)	0.99 (0.44–2.21)	0.972
**Number of IAC sessions provided**					
> 3 Sessions	207(68.1)	82 (39.6%)	125 (60.4%)	1	
3 Sessions	97(31.9)	20 (20.6%)	77 (79.4%)	1.31 (1.13–1.53)	**< 0.001**
**Duration on ART at VL non-suppression (n = 302)**					
< 1 year	62(20.5)	24 (38.7%)	38 (61.3%)	1	
> 1 to 5 years	93(30.8)	34 (36.6%)	59 (63.4%)	1.04 (0.81–1.33)	0.788
> 5 years	147(48.7)	43 (29.3%)	104 (70.7%)	1.15 (0.92–1.44)	0.209
**Time to IAC linkage**					
≤ 1 month	153(50.3)	50 (32.7%)	103 (67.3%)	1	
> 1 month- 2 month	88(29)	31 (35.2%)	57 (64.8%)	0.96 (0.80–1.16)	0.690
> 2 months	63(20.7)	21 (33.3%)	42 (66.7%)	0.99 (0.81–1.22)	0.926
**Baseline non-suppressed viral load test result (Copies/ml)**					
≥ 10,000	137(45.0)	66 (48.2%)	71 (51.8%)	1	
5,000–10,000	34(11.2)	14 (41.2%)	20 (58.8%)	1.10 (0.80–1.52)	0.557
1,000–4,999	133(43.8)	22 (16.5%)	111 (83.5%)	1.52 (1.27–1.81)	**< 0.001**
**ART regimen at time of VL non-suppression**					
EFV Based regimen	134(44.1)	57 (42.5%)	77 (57.5%)	1	
NVP Based regimen	59(19.4)	28 (47.5%)	31 (52.5%)	0.91 (0.69–1.21)	0.536
ATV/r Based regimen	35(11.5)	8 (22.9%)	27 (77.1%)	1.34 (1.06–1.69)	**0.013**
DTG Based regimen	59(19.4)	2 (3.4%)	57 (96.6%)	1.68 (1.44–1.96)	**< 0.001**
LPV/r Based regimen	17(5.6)	7 (41.2%)	10 (58.8%)	1.02 (0.67–1.56)	0.914

**Table 4:** Multivariable analysis

Characteristic	Crude RR (CI95%)	P value	Adjusted RR (CI95)	P-value
**Age in years**				
18–29	1		1	
30–39	0.92 (0.76–1.12)	0.420	0.91 (0.76–1.09)	0.305
≥ 40	0.98 (0.80–1.20)	0.830	0.88 (0.73–1.06)	0.179
**Sex**				
Female	1		1	
Male	1.15 (0.98–1.34)	0.090	1.08 (0.94–1.26)	0.285
**Number of IAC sessions provided**				
> 3 Sessions	1		1	
3 Sessions	1.31 (1.13–1.53)	**< 0.001**	1.33 (1.15–1.53)	**< 0.001**
**Baseline non-suppressed viral load test result (Copies/ml)**				
≥ 10,000	1		1	
5,000–10,000	1.10 (0.80–1.52)	0.557	1.24 (0.89–1.73)	0.200
1,000–4,999	1.52 (1.27–1.81)	**< 0.001**	1.47 (1.25–1.73)	**< 0.001**
**ART regimen at time of VL non-suppression**				
EFV Based regimen	1		1	
NVP Based regimen	0.91 (0.69–1.21)	0.536	0.91 (0.69–1.19)	0.482
ATV/r Based regimen	1.34 (1.06–1.69)	**0.013**	1.32 (1.05–1.66)	**0.017**
DTG Based regimen	1.68 (1.44–1.96)	**< 0.001**	1.61 (1.37–1.88)	**< 0.001**
LPV/r Based regimen	1.02 (0.67–1.56)	0.914	0.99 (0.66–1.48)	0.963

## Electronic supplementary material

Below is the link to the electronic supplementary material.

Supplementary Material 1 [13]

## Data Availability

Data used during this study is available upon request from the corresponding author.

## Abbreviations

IAC: Intensified Adherence Counselling
EAC: Enhanced Adherence Counseling
VL: Viral Load
ART: Anti-Retroviral Treatment
HIV: human immunodeficiency virus
WHO: World Health Organization
