# Supplementary material for: Viral load suppression after intensive adherence counselling among adult people living with HIV at Kiswa health centre, Kampala: a retrospective cohort study. Secondary data analysis
Source: AIDS Res Ther. 2023 Mar 30;20:18. doi: 10.1186/s12981-023-00513-3 (PMC10061832; doi:10.1186/s12981-023-00513-3)
Supplement: Supplementary file 1 — Supplementary Material 1 [13] [file 12981_2023_513_MOESM1_ESM.docx]

Table 5: 5As Counseling framework for adherence support for people with non-suppressed viral load

| **Guide** | **Components** |
| --- | --- |
| **IAC Session 1** | |
| **Assess** | Explain purpose of session  Disclose VL test results to client and explain the meaning of suppressed and non-suppressed VL.  Explain reasons for non-suppressed VL results (non-adherence to drugs or drugs may not be working well).  Discuss implications of non-suppressed results to the client  Determine adherence levels  Calculate the adherence score using the adherence percentage formula.  Assess client’s barriers to adherence  Use the adherence assessment checklist to ascertain client’s adherence practices.  Identify barriers to client’s adherence (arising from the assessment). |
| **Advise** | Identify information gaps from assessment  Educate client in relation to specific barriers identified  Review benefits of good adherence  Assess client’s knowledge of benefits  Provide correct and complete information  Discuss consequences of non-adherence  Assess clients knowledge on the dangers of non-adherence  Educate client on the consequences of non-adherence |
| **Assist** | Evaluate the underlying causes of the identified barriers  Prioritize the barriers  Identify possible root causes of each barrier (where applicable)  Identify client specific strategies to overcome identified barriers  Discuss possible options to address key barriers  Provide information about available support systems e.g. Community-based organizations, peer support groups etc  Discuss the pros and cons of each strategy/option |
| **Agree on** | Agree on client’s action points to address the key barriers  Identify appropriate strategies  Provide relevant and necessary information  Evaluate each action point using the 5 Ws and 1H  What, where, when, who, which , how?  Document agreed upon action points on the IAC session form  Develop and document a new adherence plan on the IAC session form |
| **Arrange** | Summarize the session  Review the action points  Review the new adherence plan  Arrange for ART refill  Explain the schedule for IAC intervention  Explain the number of sessions  Emphasize appointment keeping  Schedule the 2nd IAC session  Document the next appointment date on the IAC session form  Remind client to bring remaining pills at next visit  Refer and link to other services as appropriate |
| **IAC Session 2** | |
| **Assess** | Assess adherence levels  Document the adherence score  Compare current score with the previous  Assess progress in dealing with barriers  Identify what worked  Identify what did not work  Discuss new strategies  Assess compliance to adherence plan  Identify what worked  Identify what did not work  Discuss new strategies  Assess for possible new barriers to adherence  Use adherence assessment checklist |
| **Advise** | Do as in IAC Session 1 |
| **Assist** | Do as in IAC Session 1 |
| **Agree on** | Do as in IAC Session 1 |
| **Arrange** | Do as in IAC Session 1 |
| **IAC Session 3** | |
| **Assess** | Do as in IAC Session 2 |
| **Advise** | Do as in IAC Session 1 |
| **Assist** | Do as in IAC Session 1 |
| **Agree on** | Do as in IAC Session 1 |
| **Arrange** | Review adherence scores for 1st, 2nd and current IAC visits  If adherence score is consistently good (>95%) for three consecutive IAC visits, give 1 month appointment for 2nd VL bleeding  If adherence score is not consistently good for three consecutive IAC sessions, give appointment for 4th IAC session  Give appointment for 2nd bleeding for VL test(After 1 month)  Remind and emphasize to client to keep the next appointment.  Flag the client’s file as due for repeat VL testing ( indicate due date on the red sticker)  Discuss reminder plans with clients who are due for bleeding  Provide ARV drugs for 1 month (strictly)  Call client 1 week to the due date to remind them of appointment |
